# Extensive Characterization and Comparison of Endothelial Cells Derived from Dermis and Adipose Tissue: Potential Use in Tissue Engineering

**DOI:** 10.1371/journal.pone.0167056

**Published:** 2016-11-30

**Authors:** Hanneke N. Monsuur, Ester M. Weijers, Frank B. Niessen, Amit Gefen, Pieter Koolwijk, Susan Gibbs, Lenie J. van den Broek

**Affiliations:** 1 Department of Dermatology, VU University Medical Center, Amsterdam, The Netherlands; 2 Research Institute MOVE, Amsterdam, The Netherlands; 3 Department of Plastic, Reconstructive and Hand Surgery, VU University Medical Center, Amsterdam, The Netherlands; 4 Department of Biomedical Engineering, Faculty of Engineering, Tel Aviv University, Tel Aviv, Israel; 5 Department of Physiology, Institute for Cardiovascular Research (ICaR-VU), VU University Medical Center, Amsterdam, The Netherlands; 6 Department of Oral Cell Biology, Academic Center for Dentistry Amsterdam (ACTA), University of Amsterdam and VU University, Amsterdam, The Netherlands; Qatar University College of Health Sciences, QATAR

## Abstract

Tissue-engineered constructs need to become quickly vascularized in order to ensure graft take. One way of achieving this is to incorporate endothelial cells (EC) into the construct. The adipose tissue stromal vascular fraction (adipose-SVF) might provide an alternative source for endothelial cells as adipose tissue can easily be obtained by liposuction. Since adipose-EC are now gaining more interest in tissue engineering, we aimed to extensively characterize endothelial cells from adipose tissue (adipose-EC) and compare them with endothelial cells from dermis (dermal-EC). The amount of endothelial cells before purification varied between 4–16% of the total stromal population. After MACS selection for CD31 positive cells, a >99% pure population of endothelial cells was obtained within two weeks of culture. Adipose- and dermal-EC expressed the typical endothelial markers PECAM-1, ICAM-1, Endoglin, VE-cadherin and VEGFR2 to a similar extent, with 80–99% of the cell population staining positive. With the exception of CXCR4, which was expressed on 29% of endothelial cells, all other chemokine receptors (CXCR1, 2, 3, and CCR2) were expressed on less than 5% of the endothelial cell populations. Adipose-EC proliferated similar to dermal-EC, but responded less to the mitogens bFGF and VEGF. A similar migration rate was found for both adipose-EC and dermal-EC in response to bFGF. Sprouting of adipose-EC and dermal-EC was induced by bFGF and VEGF in a 3D fibrin matrix. After stimulation of adipose-EC and dermal-EC with TNF-α an increased secretion was seen for PDGF-BB, but not uPA, PAI-1 or Angiopoietin-2. Furthermore, secretion of cytokines and chemokines (IL-6, CCL2, CCL5, CCL20, CXCL1, CXCL8 and CXCL10) was also upregulated by both adipose- and dermal-EC. The similar characteristics of adipose-EC compared to their dermal-derived counterpart make them particularly interesting for skin tissue engineering. In conclusion, we show here that adipose tissue provides for an excellent source of endothelial cells for tissue engineering purposes, since they are readily available, and easily isolated and amplified.

## Introduction

Regenerative medicine strategies are being explored for the treatment of several pathologies, such as cardiovascular defects [1], bone defects [2,3], skeletal muscular defects [4] and difficult to heal skin wounds [5,6]. When attempts are being made to develop living tissue-engineered constructs which can be applied to a patient, a major issue in this field is that the constructs initially lack a sufficient supply of oxygen and nutrients before they become vascularized. One means of overcoming this problem is to incorporate vascular cells or a vascular network during the construction of a tissue-engineered graft [7]. For several applications in tissue engineering vascularization of the tissue is considered as a requirement for further construct development [8–12].

Skin tissue engineering is the most advanced area of tissue engineering. A number of constructs are already being used to treat large burns and ulcers, for example decellularized human dermis (Glyaderm® [13]), artificially made acellular dermal template (Integra® [14,15]) dermal substitutes containing fibroblasts (Dermagraft® [16]) and full-thickness skin substitutes (allogeneic Apligraf® [17]; autologous Tiscover® [5,18]). Although the results are very promising there is room for improvement with regards to vascularization. In all cases, graft take is reliant on fast ingrowth of new vessels (angiogenesis) once the construct is placed on the wound bed. In the case of dermal templates, vascularization of the construct is required before a split-thickness autograft can be applied on top of the dermal template [13–15]. Improving the rate of vascularization would enhance graft take and result in faster wound closure. This can be achieved by creating a prevascularized construct that restores the skin in a single step procedure [14,15,19]. Quick formation of anastomoses between vessels in the construct and recipient vessels in the wound bed avoids the slow process of angiogenesis [20,21]. The endothelial cells to be used in a construct should have a good capacity to proliferate, migrate and to form new blood vessels. Several strategies to create prevascularized constructs have been developed using either mouse endothelial cells [22], human dermal endothelial cells [21,23], human umbilical vein endothelial cells [24], human blood outgrowth endothelial cells [25] or recently with human adipose-EC [9]. In skin tissue engineering the most obvious choice is to use dermal-EC from the patient. Unfortunately, obtaining large quantities of endothelial cells from dermis is not possible in many cases, as patients with large burn wounds do not have enough viable skin left. A good alternative source for endothelial cells might be provided by the endothelial cells in the adipose-SVF, since it can be obtained from the patient by liposuction. Consequently, adipose-EC and the adipose-SVF have attracted interest for their potential suitability for tissue engineering [9,12,26–28]. However, no studies have extensively characterized the adipose-EC yet.

In this study, adipose-EC were purified, characterized and compared to donor matched dermal-EC. Cell characteristics such as surface marker expression, proliferation and migration capacity were assessed. Their angiogenic capacity was studied using a 3D fibrin matrix and the secretion of several cytokines, chemokines and angiogenic factors was determined. Our data indicates that the adipose tissue would indeed provide an excellent alternative source of endothelial cells for tissue engineering.

## Materials and Methods

### Human tissue

Human adult skin with underlying adipose tissue was obtained from healthy individuals undergoing abdominal dermolipectomy (plastic surgeon, author FBN). The discarded skin was collected anonymously if patients had not objected to use of their rest material (opt-out system). Tissue was collected from 7 donors (4 female, 3 male), age between 33 and 63. Tissue collection procedures were performed in compliance with the ‘Code for Proper Use of Human Tissues’ as formulated by the Dutch Federation of Medical Scientific Organizations (www.fmwv.nl) and following procedures approved by the institutional review board of the VU University medical center. According to the Dutch law, neither approval of an ethics committee nor written consent of the patients is required when using surgical waste material.

### Cell isolation and culture of adipose- and dermal-EC

Adipose tissue was separated from the skin, carefully removing all adipose tissue from the dermis. Adipose-tissue derived stromal cells (ASC) and dermal derived stromal cells (DSC) were isolated by collagenase type II/dispase II treatment from healthy human adult adipose tissue/skin as previously described by Kroeze *et al*. [29]. The stromal cells (a.o. fibroblasts and endothelial cells) from both tissues were cultured on flasks precoated with 1% gelatin (Sigma-Aldrich, St. Louis, USA) in DMEM (Lonza, Verviers, Belgium), 5% Fetal Clone III serum (FCIII) (Fisher Scientific, Loughborough, UK) and 1% penicillin/streptomycin (P/S) (Invitrogen, Gibco, Paisley, United Kingdom). The cells were grown at 37°C, 5% CO_2_ for approximately 3–5 days until 70–80% confluency was reached. Purification of endothelial cells from the adipose- and dermal stromal cell populations was performed by a MidiMACS separator using microbeads against CD31 (Miltenyi Biotec, Leiden, The Netherlands) following manufacturers protocol with three additional 3ml wash steps with PBS after washing with medium. The MACS separation was repeated 1–3 times until a >99% pure population of CD31^+^/CD90^-^ adipose- and dermal-EC were obtained, as confirmed by flow cytometry. The purified endothelial cells were cultured at 37°C, 5% CO_2_ on gelatin-coated culture flasks in human microvascular endothelial cell medium (= HMVEC medium) containing: M199 medium (Lonza, Verviers, Belgium), 1% P/S, 2mM L-glutamin (Invitrogen, Gibco, Paisley, United Kingdom), 10% heat-inactivated New Born Calf Serum (NBCS) (Invitrogen, Gibco, Paisley, United Kingdom), 10% heat-inactivated Human Serum (HS) (Sanquin, Amsterdam, The Netherlands), 5 U/mL heparin (Pharmacy VUmc, Amsterdam, The Netherlands) and 3,75 μg/mL endothelial cell growth factor (ECGF, crude extract from bovine brain) (Physiology department VUmc, Amsterdam, The Netherlands) [30]. The adipose- and dermal-cell populations were treated equally during the entire isolation and purification procedure. The endothelial cells were stored in the vapour phase of liquid nitrogen until required. Donor-matched endothelial cells between passage 3 and 8 were used for experiments and prior to each experiment CD31^+^/CD90^-^ (>99%) expression was confirmed on the cells by flow cytometry.

### Exposure of endothelial cells to TNF-α

Adipose- and dermal-EC were seeded in an equal density of 12x10^3^ cells/cm^2^ on gelatin-coated culture plates initially in HMVEC medium. When the cells reached 80% confluency the HMVEC medium was replaced by M199 medium, 10% HS, 10% NBCS, 1% P/S, 2mM L-glutamin (= HMEC medium). 16 h later monolayers of EC were exposed to 0, 2 or 10 ng/mL TNF-α (ReliaTech GmbH, Wolfenbuttel, Germany) in HMEC medium for 4 h, after which the monolayers were washed twice with HBSS/0.5mM EDTA and new HMEC medium was added. After 24 h, supernatants were collected for ELISA and the cells were collected for cell number quantification. Cell number was determined based on DNA content of cell pellets using CyQuant cell proliferation assay kit according to manufacturer’s specifications (C7026, Invitrogen, Carlsbad, USA). No differences were found between the number of adipose-EC and donor-matched dermal-EC.

### Proliferation assay

Basal proliferation of the adipose- and dermal-EC was followed from passage 4 to 9 on HMVEC medium. The endothelial cells were seeded on gelatin-coated culture plates in a density of 6x10^3^ cells/cm^2^ and simultaneously passaged when approximately 80% confluency was reached. The cells were passaged every 3–6 days depending on the rate of proliferation and for a total period of 18–19 days. Counting of the cells was performed in duplicate using the Accu chip and digital cell counter (Digital bio, Seoul, Korea).

Proliferation of adipose- and dermal-EC in response to angiogenic growth factors was also determined using ^3^H-thymidine incorporation, adapted from Weijers *et al*. [31]. Proliferation was studied in medium containing less serum and supplements in order to determine their response to pro-angiogenic factors basic fibroblast growth factor (bFGF) or vascular endothelial growth factor (VEGF). The endothelial cells were seeded on gelatin-coated culture plates in a density of 6x10^3^cells/cm^2^ in M199 medium with 10% NBCS and 1% P/S. Cells were left to adhere to the gelatin-coated culture plates for 16 h, followed by 72 h stimulation with either VEGF (0, 0.3, 1, 3, 7.5, 10 ng/mL; ReliaTech GmbH, Wolfenbuttel, Germany) or bFGF (0, 0.3, 1, 3, 7.5, 10 ng/mL; ReliaTech GmbH, Wolfenbuttel, Germany) in M199 medium with 10% NBCS and 1% P/S. During the last 16 h of growth, 1 μCi ^3^H-thymidine (Perkin Elmer, Belgium) was added to quantify the amount of DNA replication as measure for proliferation. The beta-emission was measured with Ultima Gold scintillation fluid on a 1900 TR Liquid Scintillation Analyzer (Packard Bioscience, Massachusetts, USA).

### Cell migration assay

Adipose- and dermal-EC were seeded in an equal density of 12x10^3^cells/cm^2^ on gelatin-coated culture plates in HMVEC medium. The HMVEC medium was replaced when the cells reached confluency by HMEC medium (containing no growth factors or heparin thus preventing EC proliferation) for 8 h before the start of the experiment. A scratch was drawn in a confluent monolayer of adipose- and dermal-EC with a plastic disposable pipette tip, after which the cell cultures were washed with M199 medium to remove detached cells. Then the cells were exposed to HMEC medium supplemented with different concentrations of bFGF (0, 0.1, 0.3, 1, 3, 10 ng/mL). Pictures of the damaged area were taken at t = 0 h and t = 16 h using phase contrast microscopy. The pictures were analyzed using an image processing algorithm by which the damaged area was measured [32]. The closed area was determined by subtracting the damaged area at time point t = 16 h from t = 0 h.

### In vitro sprouting assay

*In vitro* tube formation was studied using 3D fibrin matrices and adipose- or dermal-EC, adapted from Koolwijk *et al*. [33]. Briefly, fibrin matrices were prepared by addition of thrombin (0.5 U/mL) (MSD, The Netherlands) to a 3 mg/mL fibrinogen (Enzyme Research Laboratories, Leiden, The Netherlands) solution in M199 medium and 100 μl was added to the wells of a 96-well plate. After polymerization, thrombin was inactivated by incubating the matrices with HMEC medium. Adipose- or dermal-EC were seeded at a confluent density of 6x10^4^cells/cm^2^. After 16 h, the adipose- and dermal-EC were stimulated with HMEC medium or HMEC medium supplemented with 2 ng/mL TNF-α and VEGF (0, 0.1, 0.3, 1, 3, 10, 25 ng/mL) or bFGF (0, 0.1, 0.3, 1, 3, 10 ng/mL). The sprouts formed by adipose- and dermal-EC into the fibrin matrices were photographed and analyzed using a Nikon Eclipse 80*i* microscope and NIS-elements AR software 3.2. The amount of sprouting is expressed as surface area of the sprouts as a percentage of the total surface of the picture.

### Histological analysis

Fibrin gels were formalin-fixed and embedded in paraffin according to standard protocols. Paraffin embedded sections of 15 μm were stained with haematoxylin and eosin for morphological analysis. The sections were photographed using a Nikon Eclipse 80*i* microscope.

### Flow cytometric analysis of CD marker and angiogenic receptor expression

Receptor expression of typical endothelial markers was measured by flow cytometry at passage 3–5 for three matched donors. Cells were incubated with antibodies for 30 min at 4°C, washed with PBS supplemented with 0.1% bovine serum albumin and 0.1% sodium azide and then resuspended in the same buffer for flow cytometric analysis. The fluorescence was measured on a BD FACS Calibur and the results analyzed by Cell Quest software (Becton Immunocytometry Systems, Mountain View, CA, USA). PE-labelled antibodies and corresponding isotypes (mouse anti human) were used from BD Pharming unless otherwise stated: CD31 (WM59, IgG1), CD34 (581, IgG1), CD54 (HA58, IgG1), CD105 (SN6, IgG1, Invitrogen), CD106 (51-10C9, IgG1), CD144 (55-7H1, IgG1), CD309 (89106, IgG1), CD181 (5A12, IgG2b), CD182 (6C6, IgG1), CD183 (1C6, IgG1), CD184 (12G5, IgG2a, R&D systems), CD192 (48607, IgG2b, R&D systems), IgG1 (MOPC-21), IgG2a (G155-178), IgG2b (27–35), FcR Blocker (Miltenyi Biotec).

### Secretion of angiogenic factors, cytokines and chemokines

For the quantification of cytokines, chemokines and angiogenic factors secreted by the endothelial cells, Enzyme-Linked Immuno Sorbent Assays (ELISA) were performed using commercially available ELISA antibodies. All reagents were used in accordance to the manufacturer’s specifications. uPA, PDGF-BB, PAI-1, Angiopoietin-2, VEGF, HGF, Il-6, CXCL1, CXCL10, CXCL12, CCL2, CCL5, CCL20, CCL27 (all R&D Systems, Abingdon, UK) and CXCL8 (Sanquin, Amsterdam, The Netherlands). ELISA results are expressed as amount of angiogenic factor/cytokine/chemokine in ng/mL.

### Statistical analysis

Normality testing (D’Agostino & Pearson omnibus normality test) was performed prior to further testing. A one-way ANOVA was performed to establish significance of stimulation over the complete concentration curves as indicated in the figures by horizontal lines. To determine differences between the origins of the endothelial cells, a two-way ANOVA was executed, followed by a Sidak’s multiple comparison test. A Wilcoxon matched-pairs signed rank test was performed to analyse the flow cytometry data and a paired T-test was performed to analyse basal proliferation data. All data was obtained from three to five independent experiments with different donors in triplicate wells. In each experiment, the adipose- and dermal-EC were donor-matched. Differences were considered significant when *P<0.05, **P<0.01, ***P<0.001. Results are shown as mean ± SEM. GraphPad Prism 5 software (GraphPad Software Inc., San Diego, USA) was used to construct all graphs and tables and perform statistical analysis.

## Results

### Adipose-EC have a similar phenotype compared to dermal-EC

**Fig 1:**
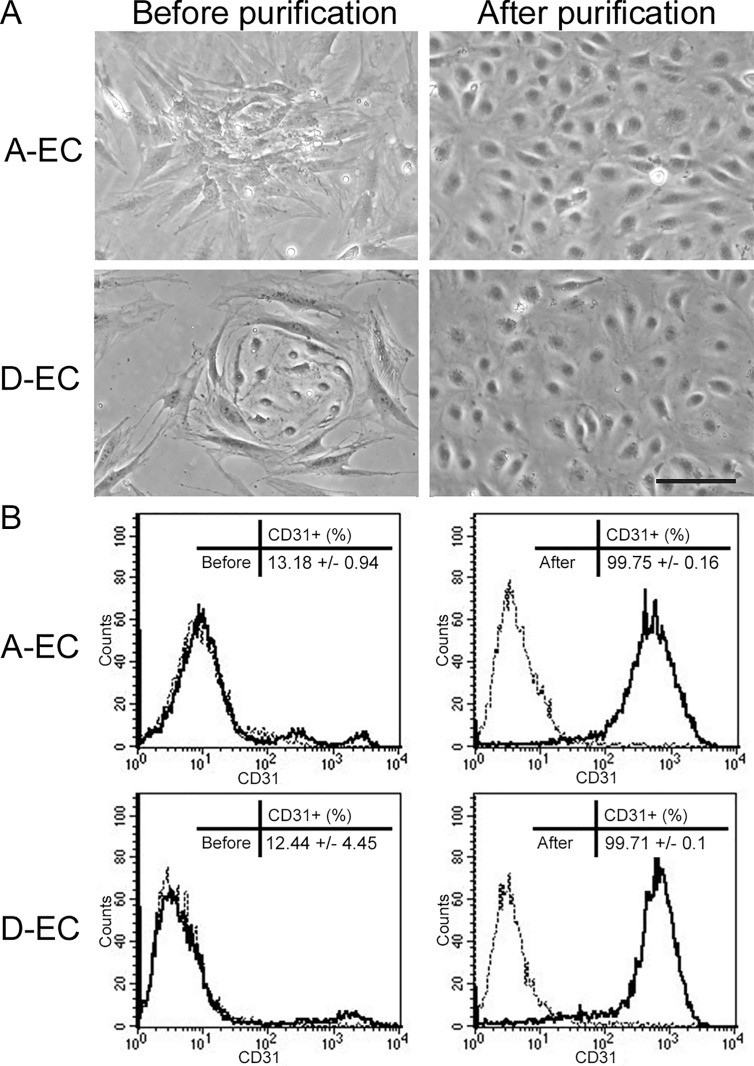
Phenotype of endothelial cells before and after purification. A) Phenotype of adipose- (A-EC) and dermal- (D-EC) EC before and after MACS purification. The scale bar represents 50 μm. B) Histogram of A-EC or D-EC before (left) and after (right) purification. Dotted line = isotype; Black line = CD31 positive EC. The insert shows mean percentage ± SD CD31 positive cells before and after purification of 4 donors (passage 3–4).

If adipose-EC are to be used as an alternative to dermal-EC in skin tissue engineering, it is important to characterize and compare both endothelial cell types donor-matched. Therefore we first determined whether both cell types have a similar expression of EC surface markers and angiogenic receptors. From 10 gram dermis, 10-20x10^6^ stromal cells were obtained and from 10 gram adipose-tissue 5-10x10^6^ stromal cells. The number of EC before purification varied per donor between 4–16% of the total stromal population. Groups of adipose-EC were often covered by ASC, while groups of dermal-EC were surrounded by DSC (Fig 1A).

After MACS selection for CD31 positive cells, a >99% pure population of EC was obtained within two weeks of culture (passage 3–4) (Fig 1B). The EC were used for flow cytometric analysis of surface biomarker expression between passage 3 and 5. Adipose- and dermal-EC expressed the typical endothelial markers PECAM-1, VE-cadherin and VEGFR2 to a similar extent with 80–99% of the cell population staining positive, whereas less than 12% of EC expressed CD34. VCAM1 was not expressed on the EC (Table 1). Since many chemokines are released at inflammation sites and are able to either inhibit or induce the angiogenic processes [34], we also determined the expression of a number of chemokine receptors (CXCR1, 2, 3, 4 and CCR2) (Table 1). With the exception of CXCR4, which was expressed on 29% of EC, all other chemokine receptors were expressed on less than 5% of the EC population. These results show no significant differences between adipose-EC and dermal-EC when cultured under identical conditions with regard to cell surface biomarker expression.

**Table 1:** Surface marker expression in cultured endothelial cells derived from human adipose tissue or dermis

Surface marker		Percentage of positive cells %				Mean fluorescent intensity			
Name	CD number	Adipose-EC		Dermal-EC		Adipose-EC		Dermal-EC	
PECAM-1	CD31	99,2	± 0,6	99,2	± 0,6	212,0	± 87,5	207,3	± 114,8
	CD34	11,5	± 12	10,1	± 5,5	0,8	± 1,3	0,6	± 0,7
ICAM-1	CD54	99,3	± 0,2	98,7	± 0,4	125,5	± 16,5	83,7	± 23,5
Endoglin	CD105	99,4	± 0,3	99,3	± 0,4	657,2	± 145,8	519,3	± 205,1
VCAM-1	CD106	1,8	± 1,6	1,3	± 1,8	0,1	± 0,2	0	± 0
VE-cadherin	CD144	99,0	± 0,3	99,1	± 0,9	71,8	± 7,7	52,2	± 14,1
VEGFR2	CD309	84,5	± 18,3	80,8	± 12,9	8,7	± 5,0	6,5	± 5,6
CXCR1	CD181	2,9	± 3,2	4,4	± 2,0	1,6	± 0	1,5	± 0,3
CXCR2	CD182	5,0	± 4,4	4,6	± 3,4	0,8	± 1,0	0,6	± 0,7
CXCR3	CD183	4,3	± 0,9	4,8	± 3,1	0,2	± 0,2	3,3	± 5,4
CXCR4	CD184	29,0	± 37,1	29,2	± 42,6	3,5	± 4,2	4,1	± 5,7
CCR2	CD192	1,2	± 0,1	0,9	± 0,4	0,4	± 0,2	0,4	± 0,1

Surface marker expression in cultured endothelial cells (between passage 3–5) derived from human adipose tissue or dermis from 3 donors (cell populations were donor matched).The mean number of positive cells for each surface marker is expressed as a mean percentage of total cell number ± SEM. The mean fluorescent intensity is expressed as the geometric mean intensity ± SEM. Differences between adipose-EC and dermal-EC were determined using a Wilcoxon matched-pairs signed rank test.

### Adipose-EC proliferate similar to dermal-EC but respond less to the mitogens bFGF and VEGF

**Fig 2:**
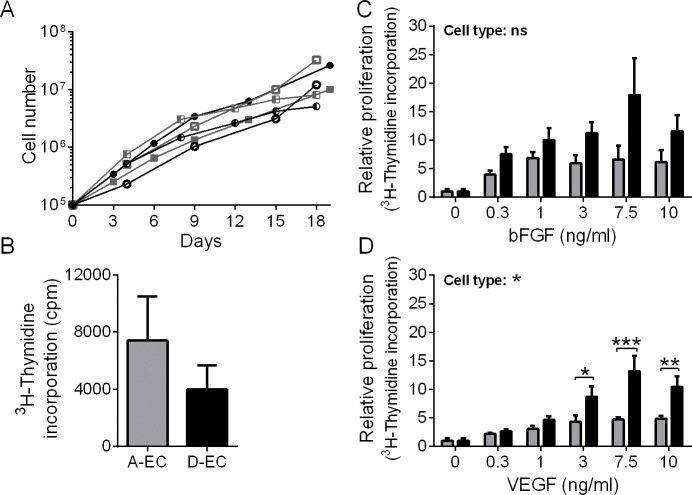
Proliferation of endothelial cells in response to bFGF or VEGF. A) Proliferation during normal culture conditions: lines of individual donors are shown, grey squares represent A-EC and black circles D-EC. B) 3H incorporation during 16h of proliferation of A-EC and D-EC in nutrient poor medium. C-D) Relative proliferation of A-EC and D-EC in response to bFGF or VEGF in nutrient poor medium. The dose-response curve to VEGF of dermal-EC shows more proliferation compared to adipose-EC (Cell type: *; two-way ANOVA followed by a Sidak’s multiple comparison test). *P<0.05, **P < 0.01, ***P<0.001. Data is shown for 4 donors as mean ± SEM. Grey bars represent A-EC and black bars D-EC. cpm = counts per minute.

During wound healing and in tissue engineering it is essential that endothelial cells are able to proliferate adequately in order to create a good vascular network. First, basal proliferation in optimal culture medium was measured for both populations (Fig 2A). The adipose- and dermal-EC proliferated equally well, although variation was observed between donors (Fig 2A). During this 18–19 day culture period EC numbers increased by approximately a 100 fold.

Next, the influence of pro-angiogenic factors bFGF and VEGF on EC proliferation as measured by ^3^H incorporation was measured during a 72 h period of culturing in nutrient poor conditions (Fig 2B–2D). A trend towards a better survival of adipose-EC than dermal-EC in unsupplemented culture medium is reflected by the ^3^H incorporation by proliferating cells (Fig 2B). When pro-angiogenic factors bFGF and VEGF were supplemented to the culture medium both the adipose- and dermal-EC showed a significant dose dependent increase in ^3^H incorporation (repeated measures one-way ANOVA, not shown). The relative ^3^H incorporation in response to VEGF was significantly lower for adipose-EC compared to dermal-EC for the higher growth factor concentrations, but not in response to bFGF (Fig 2C and 2D).

Of note, whereas the relative ^3^H incorporation was lower in adipose-EC than in dermal-EC, the absolute values of ^3^H incorporation in response to bFGF and VEGF were similar. This discrepancy between absolute and relative values is due to better proliferation under poor conditions of adipose-EC compared to dermal-EC (Fig 2B).

### Adipose-EC migrate to a similar extent as dermal-EC in a scratch assay

**Fig 3:**
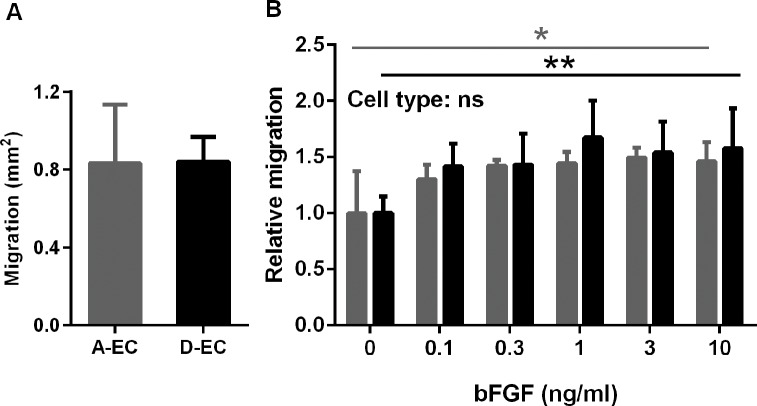
Endothelial cell migration in response to bFGF in a wound healing scratch assay. A) Basal migration of A-EC and D-EC after 16h. B) Relative migration in response to bFGF after 16h. Significance of stimulation was determined using a one-way ANOVA test. *P<0.05, **P < 0.01. Data is shown for 3–5 donors as mean ± SEM. Grey bars represent A-EC and black bars D-EC.

Since endothelial cells migrate into the wound bed during wound healing, it was determined whether differences exist in the ability of adipose-EC and dermal-EC to migrate. Migration of the endothelial cells was determined by using a scratch assay. Unstimulated adipose- and dermal-EC migrated equally fast resulting in 43.8 ± 15.8% (adipose) and 44.2 ± 6.6% (dermal) closure of the open area in 16 h (Fig 3A). Both cell types showed a similar increase in migration in response to bFGF (P<0.05) (Fig 3B). Adipose-EC closed 70.9 ± 9.9% of the open area in 16 h and dermal-EC closed 67.8 ± 4.5% when stimulated with 10 ng/ml bFGF.

### bFGF and VEGF combined with TNF-α induce sprouting of adipose-EC and dermal-EC in a fibrin matrix

**Fig 4:**
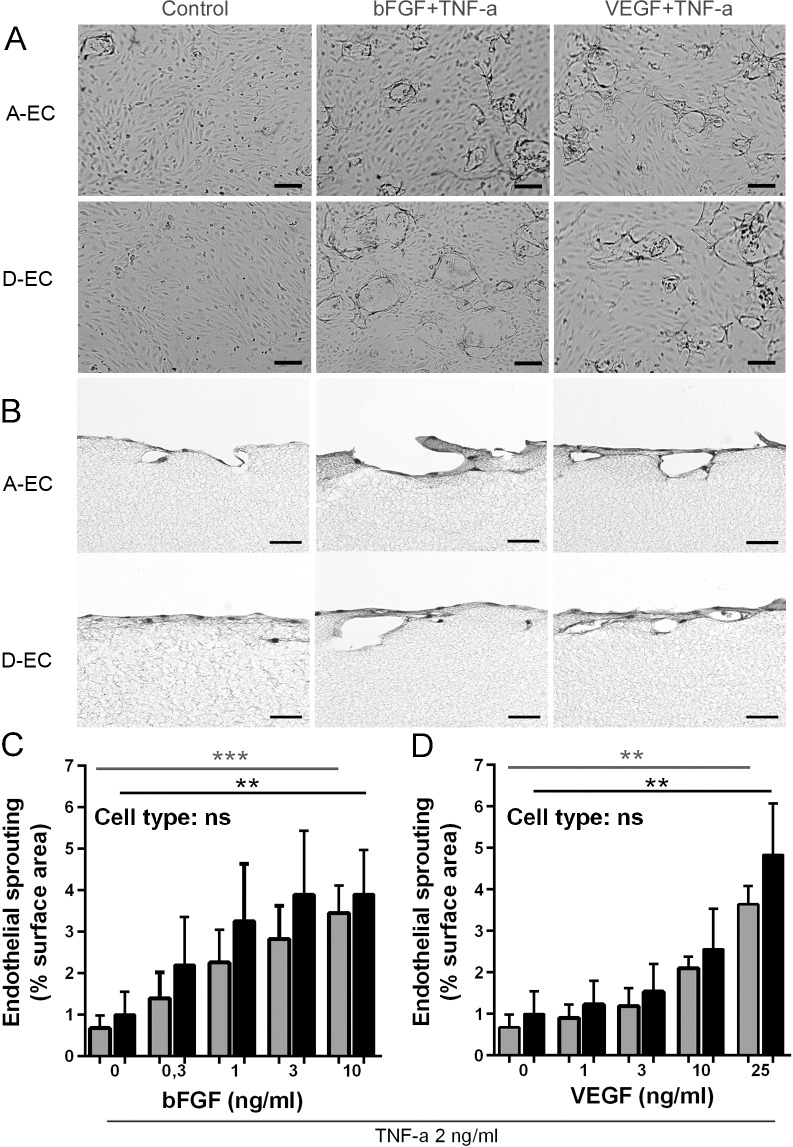
*In vitro* sprouting of adipose- and dermal-EC in a fibrin matrix. A) Surface view of A-EC or D-EC sprouting in a fibrin matrix after stimulation with HMEC medium supplemented with 2 ng/ml TNF-α alone or in combination with 10 ng/ml bFGF or 25 ng/ml VEGF. B) H&E staining of fibrin matrices with A-EC or D-EC upon stimulation with HMEC medium supplemented with 2 ng/ml TNF-α alone (Control) or in combination with 10 ng/ml bFGF or 25 ng/ml VEGF. Quantification of sprouting in response to bFGF (C) or VEGF (D). Significance of the dose response effect was determined using a repeated measures one-way ANOVA. **P < 0.01, ***P<0.001. Data is shown for 5 donors as mean ± SEM. Grey bars represent A-EC and black bars D-EC. The scale bars represent 50 μm.

New capillaries form during wound healing via angiogenesis to restore tissue vascularization. Angiogenesis takes place by the formation of sprouts from pre-existing vessels. Sprouting of EC was studied using 3D fibrin matrices cultured in HMEC medium containing TNF-α in the presence or absence of bFGF or VEGF (Fig 4) [33].

Microscopically the sprout formation was increased by both bFGF and VEGF and to a similar extent for both types of EC (Fig 4A). Both adipose-EC and dermal-EC responded strongly to stimuli, with luminized sprouts forming to a depth of approximately 50 μm within 48 h after stimulation in the dense fibrin matrix (Fig 4B). A significant dose dependent increase in sprout formation was observed for both adipose- and dermal-EC in response to bFGF and VEGF (Fig 4C and 4D).

### No differences in angiogenic factor, cytokine or chemokine secretion between adipose- and dermal-EC

**Fig 5:**
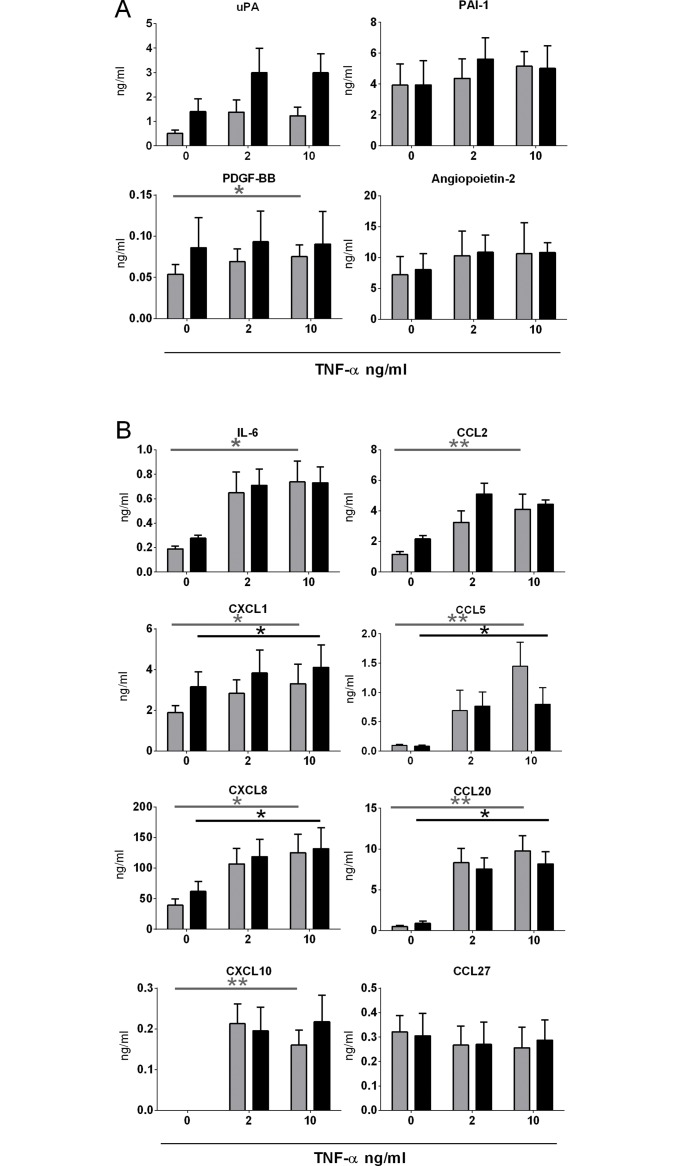
Secretion of angiogenic factors, cytokines and chemokines by endothelial cells. A) Secretion of angiogenic factors 24 h after a 4 h exposure to 0, 2 and 10 ng/ml TNF-α. B) Secretion of cytokines and chemokines 24 h after a 4 h exposure to 0, 2 and 10 ng/ml TNF-α. Significance of the dose response curve was calculated using a one-way ANOVA followed by a Dunn’s multiple comparison test. Data is shown for 4 donors as mean ± SEM. Grey bars represent A-EC and black bars D-EC.

Since endothelial cells contribute to the inflammatory phase of wound healing by secreting many angiogenic factors, cytokines and chemokines, protein secretion in response to the pro-inflammatory cytokine TNF-α was measured (Fig 5).

Of the four angiogenic factors shown, only PDGF-BB was significantly upregulated by adipose-EC after stimulation with TNF-α, and then only small amounts are secreted (Fig 5A). uPA, PAI-1 and Angiopoietin-2 were constitutively secreted by both EC. VEGF and HGF were not detected (data not shown). The secretion of the chemokines CCL5, CCL20, CXCL1 and CXCL8 was significantly upregulated by both adipose- and dermal-EC (Fig 5B). In addition, IL-6, CXCL10 and CCL2 secretion was upregulated by adipose-EC (significant) and dermal-EC (trend). CCL27 was only secreted in small amounts and not upregulated by TNF-α and CXCL12 was not detected (data not shown). No differences were observed between the two EC types for any angiogenic factor, cytokine or chemokine studied (Fig 5A and 5B).

## Discussion

Prevascularization strategies of tissue constructs have led to successful improvements in engraftment of the constructs [9,23,25–28]. Several research groups are now using adipose-EC in constructs consisting of multiple cell types, even though the properties of adipose-EC alone have not been investigated yet [9,12,26–28]. In this study an extensive characterization of adipose-EC has been performed in comparison to the widely used dermal-EC. We demonstrate here that the adipose tissue may indeed provide an excellent source of endothelial cells for tissue engineering purposes, because the adipose-EC are readily available and easily isolated and amplified. The similar characteristics of adipose-EC compared to their dermal-derived counterpart make them particularly interesting for skin tissue engineering. Of note, in our study donor matched adipose- and dermal-EC were used in all experiments. Despite some donor variation, the adipose-EC and dermal-EC perform equally well in all experiments.

It is possible to purify and obtain large quantities of EC from the adipose tissue. Similar to other studies, we found 4–16% EC within the ASC population [27,35]. The purified adipose- and dermal-EC expressed typical endothelial surface markers to the same extent, such as PECAM-1, VE-Cadherin and VEGFR2 [36]. Chemokine receptor expression on endothelial cells is important for interaction with immune cells during inflammation and for angiogenesis during wound healing. Published results on the expression of CXCR1, 2, 3, 4 and CCR2 are inconsistent as both low and high expressing cells have been reported by different research groups [37–41]. In general, endothelial cells show a clear expression of CXCR4, although there are some differences between endothelial cell types. We found CXCR4 expression on 29% of our EC, with a low mean fluorescent index. Conflicts between reports are most likely due to differences between type of EC, culture conditions and whether cell surface expression or intracellular expression was assessed [37,38,41]. In our study, in which the only variable was the cell type (adipose-EC vs. dermal-EC), no differences in surface markers were observed despite different passage numbers and donors being used.

We were able to show that both adipose-EC and dermal-EC can be easily expanded in culture, with similar isolation procedures and culture conditions. We showed an increased proliferation in response to angiogenic factors such as bFGF and VEGF. Relative proliferation in response to bFGF and VEGF revealed more proliferation of dermal-EC than adipose-EC; however the absolute amount of ^3^H incorporation in response to bFGF and VEGF was similar between adipose- and dermal-EC. This discrepancy between relative and absolute values was caused by better proliferation of adipose-EC in the nutrient poor medium (without bFGF or VEGF). The fact that adipose-EC show more proliferation under poor conditions than dermal-EC might indicate that they will also proliferate better when used to treat wounds in poor conditions.

In our scratch assay the cells were deprived from growth factors and heparin before the start of the experiment thus minimizing proliferation. Furthermore, Lauder *et al*. showed that proliferation by EC occurs after 24h, whereas our assay lasts only 16h [42]. Taken together this means that the contribution of proliferation in our assay is minimal and that the majority of the observed closure of the wound is due to migration. Both types of EC stimulated with bFGF were able to migrate quickly: ~70% closure was observed in 16 h. The speed of migration is comparable to that in studies using other types of EC [43–45]. Using HUVEC, Vitorino *et al*. found 65% closure after 15 h when stimulating with bFGF [45]. Sprouting of EC was studied using 3D fibrin matrices and was found to be strongly induced in both EC populations by addition of bFGF or VEGF in combination with TNF-α. While dermal-EC show more sprouting in response to bFGF and TNF-α when compared to adipose-EC, this result was not detected when the cells were exposed to VEGF and TNF-α. The strong induction of sprouting by the growth factors bFGF and VEGF is in line with our previous findings using dermal foreskin EC that describe a cooperative effect of TNF-α with bFGF and VEGF on EC sprouting in a fibrin matrix [33].

Endothelial cells play several roles during inflammation, including modulation of their barrier function to allow extravasation of immune cells, upregulating adhesion molecules and secreting several cytokines and chemokines. Their secretory profile differs depending on the kind of signal the cells are exposed to [46]. In this study we exposed monolayers of endothelial cells to the pro-inflammatory cytokine TNF-α. TNF-α is able to activate EC, leading to alterations in their secretory profile and surface marker expression [47–50]. The secretion of uPA, PAI-1, PDGF-BB and Angiopoietin-2 by the adipose- and dermal-EC is required during vessel sprouting, vessel growth and stabilization [51]. The adipose- and dermal-EC showed a very similar secretory profile under basal culture conditions and after activation by TNF-α. With the exception of PDGF-BB, secretion of angiogenic factors was not significantly increased. As in other studies with EC, exposure of monolayers of adipose- and dermal-EC to TNF-α resulted in increased secretion of CXCL1, CXCL8, CCL5 and CCL20 [48,50]. The proteins IL-6, CXCL10, CCL2 and PDGF-BB were significantly upregulated by adipose-EC, the secretion by dermal-EC showed a trend towards increased secretion. These results indicate that both types of EC can play a similar role during inflammation and angiogenesis. We have shown previously that a 24 h stimulation by VEGF resulted in an increased secretion of the skin-specific protein CCL27 [52]. In this study, we found that TNF-α did not stimulate CCL27 secretion. No significant differences were detected between adipose-EC and dermal-EC. The similarity in secretion of these well-known proteins gives no indication for different behaviour of adipose- and dermal-EC in wound healing.

In tissue engineering, autologous material from the patient is preferred for graft construction in order to prevent rejection. This is particularly the case when prevascularization of the graft is desired, since endothelial cells are highly immunogenic [46]. In the future, endothelial cells can be obtained in large amounts from the patient’s own adipose tissue by performing liposuction at the same time as taking biopsy material. This would make the development of a completely autologous construct feasible. Although we have concentrated on skin as the target organ, adipose-EC could equally well be used in further development of other engineered tissues and organs e.g. for cardiovascular, bone, muscle and fat tissue-engineered grafts. The ease of cell isolation and culture makes adipose tissue an ideal source of endothelial cells for easy implementation into Advanced Therapy Medicinal Products (ATMPs).

## Supporting Information

S1 Fig Surface marker expression in cultured endothelial cells derived from human adipose tissue or dermis. (PZF)

S2 Fig Proliferation of endothelial cells in response to bFGF or VEGF. (PZF)

S3 Fig Endothelial cell migration in response to bFGF in a wound healing scratch assay. (PZF)

S4 Fig *In vitro* sprouting of adipose- and dermal-EC in a fibrin matrix. (PZFX)

S5 Fig Secretion of angiogenic factors, cytokines and chemokines by endothelial cells. (PZFX)
